# Animal Welfare Assessment Protocols for Bulls in Artificial Insemination Centers: Requirements, Principles, and Criteria

**DOI:** 10.3390/ani13050942

**Published:** 2023-03-05

**Authors:** Aleksandar Cojkic, Jane M. Morrell

**Affiliations:** Department of Clinical Sciences, Division of Reproduction, Swedish University of Agriculture Sciences SLU, 75007 Uppsala, Sweden

**Keywords:** animal welfare, bulls, welfare assessment, reproduction, sperm quality

## Abstract

**Simple Summary:**

During the last 70 years, the bull semen industry has been trying to maximize reproduction efficiency to meet demands. Changes in public attitudes towards the conditions under which domestic animals are kept have led to questions being raised about animal husbandry and its impact on animal welfare. Protocols for bull welfare assessment in artificial insemination centers and how welfare disturbances can reflect on bull productivity have not previously been taken into consideration. Welfare is important for the bull industry because, apart from the known consequences of stress on reproductive parameters and performance, stress can also influence the onset of puberty and cause other health problems. Therefore, it would be useful to have an early indicator of an incipient welfare problem so that countermeasures could be taken in time to prevent such long-term effects on the animals. Different protocols have been developed for specific animal species and production groups/systems based on their biology, husbandry, management, and breeding, and care guidelines formulated. Different housing conditions, poor feeding during rearing and production, as well as poor health status have all been shown to affect bulls negatively and are reflected in sperm quality and animal fertility.

**Abstract:**

Animal welfare is a complex subject; as such, it requires a multidimensional approach with the main aim of providing the animals with the “five freedoms”. The violations of any one of these freedoms could have an influence on animal wellbeing on different levels. Over the years, many welfare quality protocols were developed in the EU thanks to the Welfare Quality^®^ project. Unfortunately, there is a lack of such summarized information about bull welfare assessment in artificial insemination stations or about how disturbed welfare can be reflected in their productivity. Animal reproduction is the basis for the production of meat and milk; therefore, factors contributing to reduced fertility in bulls are not only indicators of animal welfare but also have implications for human health and the environment. Optimizing the reproductive efficiency of bulls at an early age can help to reduce greenhouse gas emissions. In this review, welfare quality assessment will be evaluated for these production animals using reproduction efficiency as a key area, focusing on stress as a main effect of poor animal welfare and, thereby, reduced fertility. We will address various welfare aspects and possible changes in resources or management to improve outcomes.

## 1. Introduction

Modern animal husbandry relies on artificial insemination (AI) as a substitute for natural mating in some species, such as dairy cattle. One of the reasons for the introduction of AI was to control the transmission of diseases between animals. Historically, AI has offered several advantages over natural mating. The most recently advocated one is an increased rate of genetic improvement in intensive animal husbandry [1]. Other historical advantages of AI include decreasing the bull–cow ratio and decreasing the risk of bull injury compared with extensive herd breeding [2]. Since the aim of animal production is to produce meat and milk for human consumption, which is globally considered to be a One Health benefit, improving bull fertility could help to alleviate malnutrition due to insufficient proteins of animal origin [3]. However, ruminants can contribute to greenhouse gas (GHG) emissions, especially methane. Genetic improvement can help select animals with less environmental impact, improving the reproductive efficiency of these animals and thereby reducing GHG output per kg of meat or milk produced [4]. Keeping breeding bulls in Artificial Insemination Centers (AICs) decreases the number of sires that are needed to cover all the females.

One aim of holding bulls in AICs (AI bulls) is sperm production. Housing bulls in AICs varies between and within countries, but their objectives are to minimize the spreading of sexually transmitted diseases and to maximize the reproductive efficiency of the animals. Thus, a large number of males of different ages are kept in relatively static conditions at an AIC, without access to females. 

Compromised welfare can manifest in several ways, depending on the severity of the stressor and its duration of action. For example, long-term impairment of animal welfare can have profound effects on the animal, such as sub-fertility, reduced life expectancy, impaired growth, body damage, disease, immunosuppression, adrenal activity, behavior anomalies, and self-narcotization [5]. One of the key components affecting reproduction is animal welfare, which can be affected by animal housing and management, including semen collection. Therefore, it would be useful to have an early indicator of an incipient welfare problem so that countermeasures could be taken in time to prevent such long-term effects on the animals. For example, penned bulls cannot exhibit avoidance behavior by removing themselves from the vicinity of a dominant male. Changes in public attitudes towards conditions under which domestic animals are kept have led to questions being raised about animal husbandry and its impact on animal welfare. Therefore, various protocols aimed at raising welfare standards have been introduced during the past few decades. However, there is a lack of published information about factors affecting the welfare of bulls in AICs.

The concept of AICs is based on keeping male animals isolated from animals of the opposite sex. Due to the fact that breeding bulls in AICs is different than in natural conditions and that the process of semen collection is not normal reproductive behavior, requiring close human–animal interaction, there are many critical points in the semen production process that can cause animals to express inappropriate behavior. On the other hand, inadequate space and insufficient nutrition can cause health problems that further decrease animal quality of life. Therefore, the structure of this review paper is based on the main principles and requirements for welfare standards. The welfare assessment recommendations are based on indicators in the Welfare Quality Assessment Protocol for Cattle [6], which is grouped into 12 criteria based on the principles of good feeding, good housing, good health, and appropriate behavior (Supplementary Table S1). Therefore, in this review, we will discuss how disturbance of these criteria can lead to increased stress in AI bulls as a main cause of poor animal welfare, and thus lead to decreased bull semen quality. We will give a short account of stress factors and stress responses and basic bull behavior, as well as how husbandry can inhibit the expression of bull behavior and therefore act as a stressor. Then, we will summarize requirements for bulls kept as production animals (in this case, semen production) and determine objectives that are important to fulfill in order to improve welfare. 

The articles considered in this review were identified using the main search engines. The abstracts of potentially relevant articles were evaluated, and, finally, 80 articles were included and reviewed. The relevant keywords, such as animal welfare, bull welfare, poor welfare, bull reproduction, etc., were first used in the search. In the case of a lack of such literature for this specific production group, we linked it with other production groups, with animals of the opposite sex from the same species, and, eventually, with other species in a similar production group.

## 2. Main Principles, Application, and Animal Welfare Assessment

The application of animal welfare science occurs at three levels of research: (i) fundamental research that provides a basic understanding of animal welfare, (ii) research that develops ways of assessing animal welfare, and (iii) practice-oriented research, the actions that are operationalized in welfare standards or criteria [7]. Application of these levels of research can have a practical influence on animal welfare standards through requirements that contain key elements for good animal welfare. Blokhius et al. [8] classified requirements for welfare standards into three main types. First are requirements that are resource-based, which usually set out minimum standards for the animal’s environment and other resources, such as bedding, space, air quality, temperature, and access to food and water. Second are management-based requirements, which describe managers’ activities concerning animal health. They include requirements for pain management, inspecting animals and feed at a certain frequency, and having established protocols for health care and euthanasia. The final requirements are animal-based that specify the outcomes that should be achieved. These requirements include health-related markers such as maximum prevalence of lameness and injuries, allowable rates of mortality, and minimum body condition scores. In animal-based requirements, animal behavioral outcomes, focusing on low levels of aggression and stereotyped behavior, and the ability to move freely and lie down comfortably are also included [7]. These requirements were guidelines for developing different welfare assessment protocols [9].

The requirements in animal welfare standards can be viewed as serving four broad objectives [7,10], reflecting beliefs about the important factors for animals to have satisfactory welfare. The first objective is to maintain the basic health and bodily functioning of animals, reflected by a low incidence of disease and high rates of survival, reproduction, and growth. The second objective is focused on the “affective states” of animals, especially to prevent or minimize unpleasant states such as pain, distress, and hunger, and to allow animals to experience positive states such as comfort and contentment. The third objective is to provide animals with the opportunity to carry out elements of their natural behavior, especially types of behavior that animals are highly motivated to perform, such as the drive to reproduce. The final objective is to provide animals with access to natural elements in their environment such as natural light, fresh air, and the outdoors. This direct transfer of science into practice occurs especially in cases where an innovation simplifies animal management, improves productivity, or reduces production costs. If we think of animal welfare as a complex outcome that depends on a match between the genetic make-up of the animals, the production system in which they are kept, and the ability of the people to manage these animals in that system, then improving animal welfare needs to involve coordinated action in all three domains. 

These objectives were the basis for the welfare principles of the Welfare Quality^®^ (WQ) assessment protocols [6] (WAPs), created and recommended to be used by EU countries, and which we chose to follow in this review. Different WQ protocols have been developed for cattle welfare measurement on farms and in slaughterhouses, but those do not include AI bulls. The main focus for these objectives and principles evaluated in the WQ protocols was to satisfy the five freedoms point of view, which is the guiding principle that advises the World Organization for Animal Health’s work on the welfare of terrestrial animals. 

Since the beginning, the Five Domains Model [11] was developed alongside the Five Freedoms Concept [12]. The latter concept was that animals should be free from “negative” experiences (free from hunger, disease, etc.), while the Five Domains Model not only focuses on these “negative” animal welfare perspectives but also on “positive” ones. The five freedoms and five domains contain essentially the same five elements (see Supplementary Table S2). However, the five domains focus more on how an animal feels, i.e. its mental state, and distinguish between the physical and functional factors that affect its mental state. The latest updated version of the Five Domains Model [13] highlights the evaluation of negative and/or positive impacts of human behavior on animal welfare. 

Since there is little research conducted on bull welfare, specifically on AI bulls, our work in this review will be mostly based on the Five Freedoms Concept through the WQ principles and criteria. We indicate husbandry conditions for AI bulls and summarize basic knowledge of potential negative effects, mostly on animal-based requirements. However, we found the human–animal interaction in the Five Domains Model [13] to be an important assessment criterion for future research because AI bulls are animals that require close interaction with humans, due to the semen collection procedure to which the animals are regularly exposed. Therefore, we will incorporate some actions in such interactions that can promote positive welfare states. 

## 3. Animal Physiological Stress Response Due to Poor Animal Welfare

Stress appears when environmental demands exceed the regulatory capacity of an organism, particularly when an animal perceives a given situation as unpredictable and uncontrollable [14]. The two main components of the stress response are the hypothalamic–pituitary–adrenal (HPA) axis and the sympatho-adrenomedullary (SAM) system. Both plasma levels of glucocorticoids and behavior changes have been used as markers of stress. 

**Figure 1:**
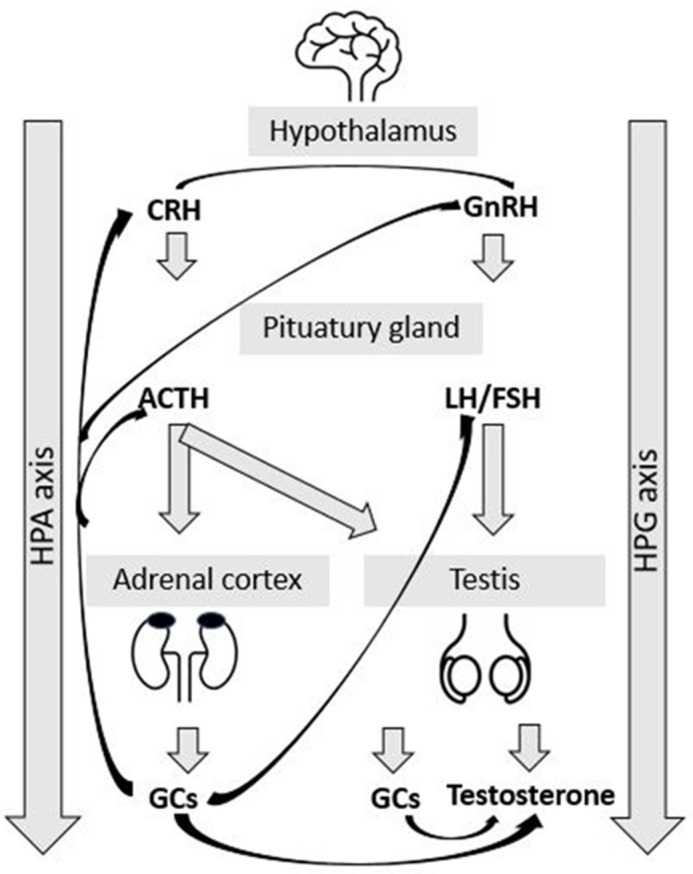
Schematic representation of hypothalamic–pituitary–adrenal (HPA) and hypothalamic–pituitary–gonadal (HPG) axis. Black arrows represent inhibitory effect of each hormone. CRH: corticotropin-releasing hormone, GnRH: gonadotropin-releasing hormone, ACTH: adrenocorticotropic hormone, LH: luteinizing hormone, FSH: follicle-stimulating hormone, and GCs: glucocorticoids.

The physiological response to stress can be described by measures of HPA axis activity. Earlier in stress research, it was assumed that increased HPA activity was a common response to all stressors, both internal and external [15]. However, Pacák and Palkovits [16] stated that there are other peripheral physiological responses to stress besides HPA axis activity. They introduced stressor-specific pathways and showed that each stressor has a neurochemical signature. This is important for further understanding of the pathogenesis and introduction of proper treatment for stress-related disorders. In the review article of Ralph et al., [17], the effects of various impacts of psychosocial stress on gonadotrophins and sexual behavior in female ruminants were discussed. Besides HPA activation, a group of neurons in the hypothalamus (arcuate nucleus) co-synthesize mediators such as kissproteins, neurokinin B, and dynorphin (KNDy cells), which interfere in the negative effect of cortisol on reproduction [17] and are also involved in the metabolic control of puberty [18]. These neuropeptides have stimulatory (kissprotein and neurokinin B) and inhibitory (dynorphin) effects on GnRH secretion regulation [19]. The neuroendocrine regulation of female reproduction starts with the synthesizing of gonadotropin-releasing hormone (GnRH) in the hypothalamus (GnRH neurons), which further stimulates gonadotrophins (luteinizing hormone (LH) and follicle-stimulating hormone (FSH)) synthesis and secretion. However, there have been claims that KNDy neurons have control over the reproductive system [19] by integrating information about the animals’ external and internal environments and influencing the release of GnRH (acting on GnRH neurons). During the stress reaction, high levels of cortisone act on KNDy cells to increase dynorphin or/and decrease kissproteins and neurokinin B. Furthermore, these changes increase the inhibition of GnRH neurons and their pulsatile secretion and eventually result in the inhibition of sexual behavior and ovulation in females [17]. The effect of this neuroendocrine regulation of reproduction has not yet been evaluated in bulls. The interaction between the HPA and the hypothalamic–pituitary–gonadal (HPG) axis is presented in Figure 1. The hypothalamus secretes corticotropin-releasing hormone (CRH), which stimulates the secretion of adrenocorticotropic hormone (ACTH) via the pituitary gland and, consequently, the synthesis and secretion of cortisol from the adrenal cortex. In cattle, as in humans, the main glucocorticoid (GC) released in stress is cortisol; it regulates the basal activity of the HPA axis and the response to stress via negative feedback at the hypothalamus in reducing CRH release. Both CRH and cortisol inhibit GnRH secretion from the hypothalamus [20,21] (cited by [22]). In addition, cortisol has a direct inhibitory effect on the secretion of LH from the pituitary gland [23] and testosterone from the testis [22,24].

Based on the duration, stress can be acute, which lasts for minutes or days, or chronic, which lasts for weeks or months (and even years) [25]. Blood sampling for cortisol or catecholamine assays may be an appropriate means of detecting HPA or SAM responses to acute stress that have a well-defined beginning and end [26]. However, this method is not reliable when dealing with prolonged stress (such as that which occurs with the effects of housing) or when the end of the stress is not well defined (e.g., where there may be prolonged effects, such as chronic pain following nose-ringing, dehorning, or tail docking) [27].

Animals subject to chronic stress generally suffer from metabolic disturbances associated with reduced feed intake, a negative energy balance, an increased metabolic rate, and, subsequently, loss of body weight or reduced growth [28,29]. On the other hand, obesity in humans leads to dysregulation of the HPG axis [30] and can result in hypogonadotropic hyperestrogenic hypoandrogenemia, a condition that affects fertility by modifying spermatogenesis, reducing testicular function, or reducing libido [31]. Although activation of the HPA axis has a wide range of effects on animals, including various pathological histological changes in a number of organs, research on cattle has concentrated mainly on documenting metabolic effects and effects on the immune system. 

Liestner and Menke [32] reviewed the literature on sex differences in the function of the HPA in humans and rodents. Preclinical models in numerous studies have observed sex differences in the stress response; thus, the HPA axis in females was activated more rapidly and produced a larger output of stress hormones than in males. However, studies with humans often produced inconsistent findings because of some associated factors, such as menstrual cycles. 

We will now consider the three main types of requirements for animal welfare standards, according to the classification by Blokhius et al. [8], in the context of AI bulls.

## 4. Resource-Based Requirements

For resource-based requirements, research often tries to identify critical levels or thresholds beyond which animal welfare indicators are affected. Such work has typically found that each production system has its specific welfare challenges and that outcomes differ considerably within the same type of system [33,34].

### 4.1. Space and Bedding (Good Housing—Comfort around Resting and Ease of Movement)

There are studies showing that space allowance and type of bedding have an influence on animal production [35,36]. To our knowledge, no studies have been conducted to explore the effects of housing conditions on the welfare and productivity specifically of breeding bulls. Mossberg [35] reviewed the welfare of growing bulls in different housing conditions. She concluded that buildings with slatted floors, and, likewise, small space allowances, or hard and slippery floor surfaces, can have a negative effect on young bulls´ health and behavior, and thus on their welfare. It was suggested that concrete and slatted floor surfaces should be softened by rubbing a coating on the slats. Animal health, especially lameness, depends on the type of floor. Haufe et al. [37] evaluated the influence of four different types of floor surface, as well as access to pasture, on claw health and concluded that the effect of floor type was slight. Likewise, access to pasture did not have an effect on floor-associated claw lesions. However, it is likely that bulls kept in AICs could reach an older age and heavier weight than the animals used in these studies, emphasizing the need for a study on AI bulls. Some AICs use sand in the semen collection area to reduce slipping. However, this can lead to accidental insertion of sand into the preputium, which later on can cause pain associated with erection and rapid loss of the mounting impulse. 

There are different housing systems for bulls in AICs, but currently, bulls are frequently kept in individual pens with access to outdoor space. The UK codes of recommendations for the welfare of cattle livestock [38], give specific recommendations for the pen size for bulls. The accommodation for a single adult bull of average size should include a sleeping area of at least 16 m^2^. For bulls weighing over one ton, the sleeping area should be at least 1 m^2^ for every 60 kg of live weight. If a bull is not regularly and routinely exercised outside the bull pen, the pen should include an exercise area at least twice as large as the sleeping area. In some countries, tethered housing was previously widely used. However, this system was shown to induce abnormal behavior in young bulls, such as abnormal licking of equipment or leaning against equipment, compared with loose-housed bulls. Sufficient bedding and/or rubber matting for stall flooring helps to prevent and/or minimize foot, leg, back, and spinal column discomfort [39].

### 4.2. Temperature and Air Quality (Good Housing—Thermal Comfort)

Since the WQ protocol for dairy cattle has no developed measure of thermal comfort, we will introduce sperm quality evaluation as a potential method based on extensive research conducted on thermal discomfort and sperm quality results. 

A high testicular temperature has an effect on sperm quality; an increased testicular temperature leads to increased production of defective spermatozoa. Physiological mechanisms to avoid an increase in testis temperature include heat loss from the scrotum via scrotal sweating, heat exchange in the testicular vascular cone, relaxation of scrotal muscles, and whole-body responses. For the production of fertile spermatozoa, testicular temperatures should be between two and six degrees Celsius lower than the bull body temperature [40]. When the testicular temperature increases, testes metabolism increases, leading to testicular hypoxia [41]. The optimal environmental temperature for sperm production varies between 15 and 18 °C during the whole period of spermatogenesis of 65 to 70 days [42]. Since spermatogenesis takes approximately 60 days, environmentally induced alterations in sperm quality might not become apparent for several weeks [43]. There are many studies reviewing the effect of increased ambient temperature on semen quality. Most of those studies include humidity as well, in the temperature humidity index (THI), and heat as prolonged heat stress, forcing animals to adapt. Berian et al. [44] evaluated the effect of heat stress (based on the THI) on the physiological and hematobiochemical profile of cattle. Increased levels of some blood parameters, such as red blood cell count, white blood cell count, packed cell volume, and hemoglobin, were followed via an increase in levels of cholesterol, creatinine, alanine transaminase (ALT), aspartate aminotransferase (AST), cortisol, and blood urea nitrogen (BUN). Physiological parameters such as respiratory and heart rate were also observed as significantly raised with an increased environmental temperature. This indicates that the THI is a sensitive indicator of heat stress. Furthermore, differences between bull species (*Bos taurus* and *Bos indicus*) were noted. *Bos taurus* bulls are more sensitive to high ambient temperatures than *Bos indicus* bulls. Even the semen quality parameters of crossbred bulls (*Bos taurus* × *Bos indicus)* recover more rapidly after heat stress than for *Bos taurus* bulls. Morrell [43] indicated that differences in temperature between seasons might be equally important, in the short term, inducing heat stress in animals with consequences on sperm quality. Therefore, providing bulls with comfortable stalls that are heated or cooled to maintain a temperate environment will positively affect sperm output, sperm quality, and bull well-being [45]. 

On the other hand, few studies have been conducted to explore the influence of air pollution on fertility in farm animals. There are systematic reviews that link the impact of air pollution with reproduction in women [46] and laboratory animals [47], as well as fertility and spermatogenesis in humans [44,48]. In the majority of the studies, reproductive and fertility parameters were negatively affected by air pollutants. Good air quality is affected by numerous factors, such as gases, dust, and microorganisms, and the management of an intensive animal breeding system that includes stocking density, the size of the cattle, flooring, bedding, waste management, building design, and ventilation system [49]. Similar studies on the effects of these pollutants on bull fertility are needed. 

### 4.3. Access to Food and Water (Good Feeding)

Access to clean drinking water and a diet that is appropriate to a species and can keep them healthy is one of the five freedoms and therefore needs to be assessed to meet animal welfare demands. 

It is known that stress influences feed intake in animals, but there are few reports on how feeding time and frequency influence animal welfare by causing a stress response. Calamari et al. [50] conducted a study on the influence of frequency and feeding time on metabolic condition and milk production in heat-stressed dairy cows. The animals were divided into three groups, and a total mixed food ration was delivered either once daily in the morning or evening, or twice daily in the morning and evening. Different plasma metabolites were analyzed, and rectal temperature and respiratory rate were noted. The results showed that feeding once daily, especially in the morning, was less suitable to provide adequate cow welfare during the hot season than the other feeding regimens. In detail, plasma glucose levels were lower in the morning feeding routine, while plasma urea was lower in the evening feeding routine. Rectal temperature and respiratory rate values were higher in the group of cows receiving food just in the morning. Milk production was not affected by feeding management. The bull husbandry requirements for optimum productivity may be different from those of dairy cows; therefore, the feeding management and production performance of AI bulls needs to be evaluated.

Recent studies on the nutritional control of puberty in female cattle demonstrate that maternal nutrition during gestation can induce morphological and functional changes in the hypothalamus system, which can persist long after the birth of female offspring, influencing reproductive performance even in adulthood [18]. Furthermore, the hormone leptin was shown to be linked to metabolic status and puberty, acting through arcuate nucleus neurons, transducing the nutritional signal into input (influencing GnRH neurons) in sheep and other species [51]. The fertility of breeding bulls is also a key factor in sustainable cattle production; proper management and nutrition of the bulls are essential to maximize reproduction efficiency [2]. Feed restriction in young bulls altered the onset of puberty in relation to plasma insulin-like growth factor-I (IGF-I) and IGF-binding proteins in [52]. The study showed a progressive increase in IGF-I in a group of bulls fed a balanced diet compared with two groups with restricted feeding, which did not show changes in IGF-I levels. The levels of IGF-binding proteins varied between groups depending on the laboratory methods that were used: radioimmunoassay (RIA) or Western ligand blotting (WLB). Previous studies by the same group of authors showed an association between puberty and plasma testosterone, insulin-like growth factor-I (IGF-I), and IGF-binding proteins [53,54]. Insulin-like factor 3 (ILF-3) is one of the peptide hormones (a member of the IGF family) that is produced mostly by Leydig cells in mammals [55], together with testosterone, and is another important reproductive hormone. This hormone was found to be crucial for testicular descent [56] but also the survival of rete germ cells [57], and recently, low levels of ILF-3 were associated with malnutrition in children [58]. The serum concentration of ILF-3 increases from birth to puberty when it reaches a plateau [59]. In adulthood, ILF-3 secretion depends on the differentiation stage of Leydig cells, which are dependent on LH [60]. However, the regulation of ILF-3 secretion during puberty differs from that of testosterone, which is regulated by the HPG axis as well as LH [59]. Therefore, serum concentrations of ILF-3 can be used as a biomarker of testis status, especially the status and number of Leydig cells. Similar findings were reported in a study on bulls [61].

## 5. Animal-Based Requirements

For many animal-based measures, such as the proportion of lame or injured animals, there are no non-zero values where welfare is not affected [8]. Assessing behavioral and health indicators as a main animal-based requirement of animal welfare can be used indirectly for resource-based and management requirements [5].

### 5.1. Health-Related Measures (Good Health)

Absence of pain is one of the main principles of good animal welfare, as well as absence of illness. There are two groups of markers of farm animal welfare that can be measured and that are health-related. The first group comprises physical indicators including cut injury, body damage, abscess formation, swelling of the joints, and loss of hair or wool. The other group consists of physiological indicators, which include cortisol level, reduced feed intake, immunosuppression, adrenal activity, and altered physiological responses, e.g. depressed reproductive parameters. Health indicators included in the welfare assessment of bulls in AICs have not been described. Physical indicators modified from the protocol for dairy cows [62] are presented in Table 1; these indicators might be used for bulls as well. 

The Office International des Epizooties (OIE) Terrestrial Animal Health Code, which implements improvement standards for animal and public health and animal welfare from a veterinary viewpoint, summarized general considerations and conditions applicable to AICs and to breeding bulls. Instructions for general hygiene in AICs are provided in Chapter 4.5 of the Health Code, although the requirements that bulls should fulfill before entering AICs are based only on potentially pathogenic infectious agents. Furthermore, recommendations are given that would reduce semen contamination with common microorganisms. However, additional details are needed, which could include an assessment of welfare biomarkers, if sufficiently sensitive indicators could be identified. 

The study by Cojkic et al. [63] indicates that differences in the seminal microbiota of healthy bulls occur and might be correlated with fertility. The influence of bacteria on fertility may depend on their effect on sperm quality parameters during storage, and also on their interaction with other bacteria. Some bacteria decrease sperm quality parameters directly or indirectly, negatively influencing fertility [63]. Therefore, sperm quality evaluation could be a good indirect parameter of bull welfare. Identification of health problems, even at the individual level, can be a useful indicator of a problem at the herd level as well. Early diagnosis of diseases is important for welfare based on their association with negative experiences such as pain, distress, and discomfort [5]. 

Excellent hoof condition is one of the most important physical aspects of pain-free semen collection. Lameness, whether from sole abscesses, foot rot, interdigital dermatitis, hoof cracks, laminitis, improper hoof trimming, etc., will often result in bulls being reluctant to mount. Furthermore, lame bulls often overcompensate for the foot pain and exert abnormal pressure on other bone and muscle groups, placing the bull at risk for greater injury [38].

### 5.2. Bull Behavior and Libido Measurements (Appropriate Behavior)

Puberty and social and sexual development in bulls are under strong genetic regulation. There are breed differences, as well as age differences, in the beginning of the manifestation of, and changes in, sexual behavior [64]. Young calves already display aspects of sexual behavior by mounting during play. Sexual behavior in bulls is differentiated into two components: libido (sexual drive) and mating ability [65]. Both can be negatively influenced by external factors, particularly stress. 

Rearing calves to become bulls in Artificial Insemination Centers (AICs) is quite unlike a situation in the wild or under extensive farm husbandry conditions. In the wild, or under extensive farm husbandry conditions, the social ranking of bulls is based on size and age within the group [66]. In a herd with multiple bulls, all tend to mate with the same sexually active cows. It is recommended to group the bulls together before the breeding season to allow them to determine their social ranking before intensive mating occurs, thus avoiding stress and possible injuries due to competition between bulls. 

There are certain behaviors that indicate a problem with animal welfare. Some of these include an increased respiratory rate or panting, decreased feed intake, and demonstration of aggressive, depressive, stereotypic, and/or other abdominal behaviors [49]. Physiological stress leads to hormonal imbalance and contributes to infertility by suppressing the hypothalamic–pituitary–ovarian axis (HPO) in rats. The mechanism of inhibition is through the inhibition of GnRH secretion, thereby suppressing LH release from the pituitary [67]. In the same study on acute stress, significant elevations in corticosterone, plasma adrenocorticotropic hormone, prolactin, and progesterone were observed, as well as alterations in sexual behavior, decreased FSH, and immunoreactive inhibin. In a similar study in men, psychological stress decreased serum testosterone levels, leading to a secondary rise in LH and FSH. Serum LH and FSH levels were negatively correlated with sperm count and positively correlated with abnormal sperm motility and morphology [24]. On the other hand, a positive correlation between serum testosterone concentration and expression of sexual behavior was noted for young and adult bulls, although the relationship was not significant [68]. However, expression of sexual behavior and serum testosterone levels were significantly higher in adult bulls compared with the younger group. 

In the study by Lange et al. [69], a good human–animal relationship had a positive influence on heifers even during restraint. The results of gentle interaction were reflected in physiological parameters by decreasing heart rate and via behaviors such as longer durations of neck stretching. However, similar studies have not been conducted for bulls. During bull semen collection, personnel actively lead the animals. Therefore, previous positive experiences and a lack of fear of humans might improve libido during sexual stimulation prior to collection. Change of personnel can have a negative effect on semen quality or, on the other hand, may help to resolve a problem, if there are no other underlying causes. Apparently, similar environments may be managed differently by a stock person, further affecting animals’ experience of a particular situation [6]. Mellor et al. [13] provided some examples that can generate positive effects in animals. The companionable presence of persons who provide feelings of safety and preferred foods, with tactical contacts and/or reinforcements, results in an increased score during welfare assessment. Furthermore, personnel who participate in enjoyable routines and engaging activities initiate a positive reaction in bulls; the calming presence of familiar persons in threatening circumstances, and taking action to end periods of deprivation, inhibition, and harm, all help to improve animal welfare. These positive impacts of interaction can be graded separately according to the frequency, variety, duration, and form of congenial contact. 

The frequency of semen collection is positively correlated with bull reaction time and novelty [39]. Management-based semen collection procedures should be reviewed for an individual bull if production goals are not met. If the bull does not show a response to stimulation in 5 min, a change in stimulus should be made [70]. One of the most effective approaches for sexual preparation is false mounting. Allowing three false mounts, followed by a final fourth mount for semen collection, was found to maximize sperm harvest. Furthermore, false mounting in combination with active restraining increased sperm concentration per ejaculate. However, these mounting opportunities need to be reduced for bulls with low libido and physical limitations.

## 6. Management-Based Requirements

For management-based requirements, science has been used in two main ways. One is simply to assess how animal welfare is affected by management practices, and the second application is to develop and test practices that improve animal welfare. For example, in the “Welfare Quality” project, a large cooperative research program that developed standards for cattle, pigs, and chickens, the scoring systems rely heavily on animal-based measures such as body condition, lameness, lesions, and agonistic behavior (e.g., Welfare Quality, 2009). Such results contribute to a growing recognition that good animal welfare is a complex outcome of different factors including animal genetics, management, and environment. 

During the last 70 years, efforts in the bull semen industry have been made to maximize reproduction efficiencies to meet demands. For this to be achieved, there is a need for management schemes to be efficient to exploit bulls’ reproductive potential and, at the same time, to minimize the risk of injury associated with it. At present, bulls younger than 15 months old are commonly included in intensive semen collection [71]. However, a comprehensive study of lifetime productivity has not been carried out for these young bulls.

Management requirements should be evaluated based on welfare principles and criteria. The welfare quality assessment protocol for cattle [6] summarized four main welfare principles and provided criteria that can be used even for the welfare assessment of bulls in insemination centers. 

Antimicrobial use is one of the management requirements in the process of semen preservation since the antibiotics are used without previous bacterial identification and testing of antimicrobial susceptibility. The sources of bacterial contamination in bull semen are the animals themselves, their environment, and the process of semen collection and preservation. The European Union provides government directives on the use of antibiotics in semen for international trade [72], aiming to avoid the transmission of potential pathogens and disease outbreaks. This non-therapeutic use of antibiotics may increase the risk for antimicrobial resistance (AMR). Currently, the Food and Agriculture Organization of the United Nations (FAO), the World Health Organization (WHO), and the Office International des Epizooties (OIE) have created the “One Health” concept with transdisciplinary public policies to minimize AMR and promote responsible antibiotic use through the aspects of human, animal, and environmental health. Albernaz-Gonçalves et al. [73] linked antibiotic use in pig farming with animal welfare. They summarized the major disadvantages of husbandry procedures used in intensive pig farming that lead to stress and that are consequential to diseases and increased use of antibiotics. They emphasize that the adoption of good management practices based on animals’ holistic needs is essential for the rational use of antibiotics. A similar approach is now needed in the bull breeding industry.

## 7. Discussion

The main aim of this review was to summarize the critical points in bull semen production where animal welfare could be compromised. Furthermore, we explained how insufficiently welfare-oriented production criteria lead to stress and, subsequently, decreased reproduction efficiency. 

Assessment of animal welfare can be challenging due to its complexity and multifactorial nature. Poor animal welfare can be easily missed due to a lack of knowledge of the species and insufficient evaluation. This can be avoided by using appropriate methods for animal welfare assessment. Two primary methods which can be used are (1) objective evaluation by scoring specific criteria and (2) use of subjective judgments and evaluation. There are differences in the scoring systems between different countries [74,75], which should be normalized to enable effective comparisons to be made. There are also differences in welfare assessment based on the animals used in different programs, for example, animals used in research programs versus those in agricultural production programs. For animal agriculture programs, six basic assessment categories were given: performance standards (animal-based measurements or outcome criteria), prohibited practices, input-based standards (engineering or design standards), five freedoms, record-based standards, and subjective evaluation. These standards can be used as a template for other animal programs and for different animal groups within the same program. 

The development of systems to evaluate animal welfare was based on scientific interest as well as public concern about the raising conditions and treatment of animals. The first versions were developed for animals used in research, teaching, and testing (RTT). In subsequent years, questions were raised about other animals and their keeping conditions. Thereafter, hundreds of welfare assessment protocols were developed around the world; all differ from one another based not only on the animal species and production group but also on the place where the animals are kept at the time of evaluation. 

Since AI technology is mainly used in the dairy industry, dairy bulls are usually the cattle kept in AICs. Krueger A et al. [9] summarized the similarities and differences between different welfare evaluation programs for dairy cattle on farm levels used in three continents: the European Welfare Quality^®^ Assessment Protocol dairy cattle (WQ), the U.S. National Dairy Farmers Assuring Responsible Management Program (FARM), and the New Zealand Code of Welfare: Dairy Cattle (The Code). The fourth, upcoming program, the Integrated Diagnostic System Welfare (IDSW), was also discussed. Some of them are voluntary (FARM, WQ, and IDSW), and some of them are required to be followed by law (The Code). Some are more animal-oriented (WQ), and others include environmental and management-based requirements (FARM, The Code, and IDSW). Looking at all of them from the bull perspective, just two of them include bull breeding (FARM and The Code), and only one protocol includes reproduction data (IDSW) for the welfare evaluation but is not complete. The FARM program includes bull breeding, based on the fact that all bulls being raised as dairy steers should be castrated, and how the procedure should be performed, with the remainder being on pain management. On the other hand, The Code gives more recommendations about natural service bulls, regarding the number of cows that needs to be served, nutrition, and environmental requirements. Some of them can be implemented for AI bulls, such as the size of the sleeping and exercise area requirement, but the rest cannot, based on the environmental conditions that animals are kept in and the level of social interaction between humans and other animals. Animals experience the same environment in different ways, based on their genetics, early experiences, and temperament [6]. 

Bulls kept in AICs are unique in many ways; they are mostly dairy breeds with specific metabolic requirements due to the intensive selection for milk production. Furthermore, husbandry conditions for AI bulls differ slightly between countries at present but have huge differences compared with other animals from the same species and between production groups (beef and dairy). In addition, semen collection is not a natural way of expressing reproduction behavior. All of these differences and specifics require detailed knowledge of AI bull physiology, behavior, and husbandry before defining criteria for welfare assessment. It might be possible to create a unique WAP for the specific conditions of AI bulls by analyzing different existing welfare assessment protocols and approaches to welfare assessment. 

Subjective evaluation is difficult to eliminate in total because of the observers´ projection of their own impression. This can be minimized by the personnel responsible for these assessments attending training programs and by establishing criteria that define good welfare customized to the species, sex, age of animals, and production purpose. The whole goal of assessment programs should focus on minimizing the subjective component. One objective assessment system, which has been developed during the past few decades, was Welfare Quality^®^ designed to determine the welfare status of cattle, pigs, and poultry in animal units. However, creating such an assessment system for other animal types, in our case for bulls in AICs, has its difficulties due to the multidimensional and multidisciplinary approach of animal welfare [76]. 

The focus of all further assessment protocols should be the same, i.e., reducing stress in animals from the beginning but also promoting positive stimulants. The reason why this demand is important for the bull industry is that, apart from the known consequences of stress on reproductive parameters and performance, stress can also influence the onset of puberty [77]. Delayed puberty increases the production time for highly genetically valuable bulls and indirectly contributes to increased greenhouse gas emission in terms of methane and nitrous oxide [78] and, as such, is implicated in the One Health concept. Currently, welfare regulations stipulating the conditions that apply to bulls kept in AICs are lacking. The current regulation [79] of conditions that apply to the keeping of calves confined for rearing and fattening can be applied to a certain point to male calves that are going to be used for semen collection. With the onset of puberty, the rearing conditions for young bulls require more consideration due to the beginning of social ranking within the group at the same time. The major factor influencing social ranking in the wild or under extensive farming conditions is bull seniority [80], and then dominance is controlled by the size of the bull. However, there is little scientific evidence on how rearing groups of bulls, of the same age and similar in size, in the same pen influences animal welfare and, consequentially, fertility results. In multiple-sire breeding groups, all bulls tend to breed with the same sexually active cows, which increases the risk of rivalry. This can be avoided by introducing young bulls into a group of mature bulls to establish the social ranking prior to turning them out into the herd containing sexually active females [2].

## 8. Conclusions

There is a lack of information on bull welfare assessment in AICs or on how disturbed welfare can be reflected in bull productivity. Production systems for breeding bulls should be described and evaluated to meet the standards of good animal welfare. More research is needed on welfare throughout an animal´s life, i.e., from when calves are selected to become breeding bulls to the end of the bulls’ productive lives. Furthermore, specific protocols for welfare quality evaluation for this specific production group of animals should be created, focusing on their physiological and behavioral needs, with the aim of achieving optimal productivity.

## Figures and Tables

**Table 1:** Health indicators, which might be included in bull welfare assessment (modified from Rousing et al. [62]).

Body Part	Clinical Parameter	Welfare Relevance	Bull Welfare Principle to Apply
General appearance	Body condition score	Poor body condition may cause long-term discomfort and an increase in disease susceptibility because of impaired immune competence; it indicates metabolic disorders, sub-optimal management, or chronic coping difficulties.	Good feeding Good health
Skin	Skin parasites Skin infection pressure sores	Pruritic skin disorders result in long-term discomfort and increase the risk of secondary self-inflicted lesions. Skin injury and infection cause acute and chronic pain. Provides information about problems regarding housing system, management, or underlying diseases.	Good housing Good health
Legs	Lameness Hoof care	Lameness indicates a painful leg condition and affects the freedom of movement and the performance of behavior. Overgrown or deformed hooves might indicate foot disorders causing pain and discomfort. The resulting changes in leg conformation might evolve into chronic articular damage.	Good housing Good health Appropriate behavior
Systemic diseases	General condition Clinical diseases	Clinical diseases typically involve pain and discomfort. The welfare implications vary according to the intensity and duration of the disease condition and welfare. General condition is affected.	Good feeding Good housing Good health Appropriate behavior
Mortality	Case history of culled animals	This information points out specific problem areas in the herd and provides details for tackling serious health problems.	Good feeding Good health

## Data Availability

Not applicable.

## Supplementary Materials

The following supporting information can be downloaded at: https://www.mdpi.com/article/10.3390/ani13050942/s1, Table S1: Principles and criteria that are the basis for the Welfare Quality® assessment protocols; Table S2: Five Freedoms and Five-Domain Model.
