# Supplementary material for: Animal Welfare Assessment Protocols for Bulls in Artificial Insemination Centers: Requirements, Principles, and Criteria
Source: Animals (Basel). 2023 Mar 5;13(5):942. doi: 10.3390/ani13050942 (PMC10000089; doi:10.3390/ani13050942)
Supplement: Supplementary file 1 [file animals-13-00942-s001.zip › animals-2220935-supplementary.pdf]

Supplementary Table S1. Principles and criteria that are the basis for the Welfare Quality® assessment protocols

Welfare principles	Welfare criteria
Good feeding	Absence of prolonged hunger<br>Absence of prolonged thirst
Good housing	Comfort around resting<br>Thermal comfort<br>Ease of movement
Good health	Absence of injuries<br>Absence of disease<br>Absence of pain induced by management procedures
Appropriate behavior	Expressing of social behaviors<br>Expressing of other behaviors<br>Good human-animal relationship<br>Positive emotional status

Supplementary Table S2. Five Freedoms and Five-Domain Model

Five Freedoms	Five Domains
1. Freedom from hunger and thirst	1. Nutrition
2. Freedom from discomfort	2. Environment
3. Freedom from pain, injury and diseases	3. Health
4. Freedom to express normal behavior	4. Behavior
5. Freedom from fear and distress	5. Mental state
